# Rapamycin Added to Diet in Late Mid-Life Delays Age-Related Hearing Loss in UMHET4 Mice

**DOI:** 10.3389/fncel.2021.658972

**Published:** 2021-04-07

**Authors:** Richard A. Altschuler, Lisa Kabara, Catherine Martin, Ariane Kanicki, Courtney E. Stewart, David C. Kohrman, David F. Dolan

**Affiliations:** ^1^Kresge Hearing Research Institute, Department of Otolaryngology, Head and Neck Surgery, University of Michigan, Ann Arbor, MI, United States; ^2^VA Ann Arbor Health Care System, Ann Arbor, MI, United States; ^3^Department of Cell and Developmental Biology, University of Michigan, Ann Arbor, MI, United States

**Keywords:** rapamycin, age-related hearing loss, aging, auditory, cochlea, deafness

## Abstract

Our previous study demonstrated rapamycin added to diet at 4 months of age had significantly less age-related outer hair cell loss in the basal half of the cochlea at 22 months of age compared to mice without rapamycin. The present study tested adding rapamycin to diet later in life, at 14 months of age, and added a longitudinal assessment of auditory brain stem response (ABR). The present study used UMHET4 mice, a 4 way cross in which all grandparental strains lack the Cdh23^753A^ allele that predisposes to early onset, progressive hearing loss. UMHET4 mice typically have normal hearing until 16–17 months, then exhibit threshold shifts at low frequencies/apical cochlea and later in more basal high frequency regions. ABR thresholds at 4, 12, 24, and 48 kHz were assessed at 12, 18, and 24 months of age and compared to baseline ABR thresholds acquired at 5 months of age to determine threshold shifts (TS). There was no TS at 12 months of age at any frequency tested. At 18 months of age mice with rapamycin added to diet at 14 months had a significantly lower mean TS at 4 and 12 kHz compared to mice on control diet with no significant difference at 24 and 48 kHz. At 24 months of age, the mean 4 kHz TS in rapamycin diet group was no longer significantly lower than the control diet group, while the 12 kHz mean remained significantly lower. Mean TS at 24 and 48 kHz in the rapamycin diet group became significantly lower than in the control diet group at 24 months. Hair cell counts at 24 months showed large loss in the apical half of most rapamycin and control diet mice cochleae with no significant difference between groups. There was only mild outer hair cell loss in the basal half of rapamycin and control diet mice cochleae with no significant difference between groups. The results show that a later life addition of rapamycin can decrease age-related hearing loss in the mouse model, however, it also suggests that this decrease is a delay/deceleration rather than a complete prevention.

## Introduction

Age-related hearing loss (ARHL) occurs in approximately one-third of people in the United States over the age of 65 increasing to approximately half of those over the age of 75 (e.g., Gates, 2006; Gates et al., 2010). ARHL can reduce ability to communicate, quality of life and social integration and has been identified as a major risk factor for depression and dementia (e.g., Gates et al., 2010; Lin et al., 2011; Davis and Smith, 2013). One major cause of ARHL is an age-related loss of sensory hair cells, predominantly outer hair cells, and an accompanying decrease in auditory sensitivity as measured by threshold shifts (TS) in auditory brain stem response (ABR) in animal models and audiometric thresholds in people. The underlying mechanisms responsible for age-related hair cell loss remain unknown and there are no treatments currently being clinically applied to prevent or reduce this pathology.

The National Institute on Aging Intervention Testing Program (NIA-ITP) tests for treatments that can be added to diet to increase lifespan, using UMHET3 mice, a four-way cross, as a model. Four-way cross mice (from four different grandparent strains) provide for genetic heterogeneity and reduce strain specific effects. Among several effective treatments identified through NIA-ITP, addition of rapamycin to diet at 9 months of age was found to increase life span by 26% in male mice and 23% in female mice (Miller et al., 2014). We hypothesized that ARHL might share underlying mechanisms, such that treatments that enhance life span could also reduce and/or delay ARHL. This is consistent with studies that demonstrate the positive effects of rapamycin on age-related disorders in animal models, including decreases in cardiac pathology (Dai et al., 2014); muscle weakness (Bitto et al., 2016), cancer incidence (Anisimov et al., 2011), and cognitive decline (Halloran Hussong et al., 2012; Majumder et al., 2012).

In a previous study (Altschuler et al., 2018) we evaluated cochleae from 22 months old UMHET3 mice that had rapamycin added to their diet at 4 months of age as well as from control littermates with normal diet. The 22 months old rapamycin-treated mice had significantly less loss of outer hair cells in the basal half of the cochlea compared to the untreated controls. This sparing of hair cell loss was restricted to the basal half of the cochleae, while the apical half of the cochleae exhibited equivalently large losses of outer hair cells in both rapamycin-fed and normal diet controls (Altschuler et al., 2018).

These results showing rapamycin could reduce or delay age-related hair cell loss in the basal half of the cochlea at 22 months of age raised two pertinent issues. First, the apparent limitation of the treatment effect of rapamycin to the basal half of the cochlea could reflect differences in mechanisms underlying hair cell loss along the cochlear spiral. Alternatively, the effect of rapamycin treatment could be due to delaying rather than preventing age-related hair cell loss. Hair cell loss occurs earlier in apical vs. basal cochlea in most mouse strains (for reviews; Gratton and Vazquez, 2003; Ohlemiller, 2006). If the effect of rapamycin is to “delay” rather than prevent age-related hair cell loss, a treatment induced difference in apical cochleae might also have been present at an earlier time, but by 22 months of age the delay was over and the hair cell loss had equilibrated. The current study was designed to address these points by generating a longitudinal assessment of auditory brain stem response (ABR) thresholds at 4, 12, 24, and 48 kHz in individual mice at 5, 12, 18, and 24 months of age. The current study also addressed a second question of whether beginning rapamycin treatment at a more clinically relevant later age would still be effective in reducing or delaying ARHL. Recent studies have shown that rapamycin can extend life span in mice even when added to diet at 19–20 months of age (Harrison et al., 2009; Zhang et al., 2014) and can also reduce age-related pathologies such as cancer incidence and decreased muscle (Zhang et al., 2014) and cardiac function (Quarles and Rabinovitch, 2020) with late life application. We therefore chose to add rapamycin at a later time but prior to the first appearance of ARHL. Three of the four “grandparent strains” of the UMHET3 mice carry homozygous *ahl* alleles (*Cdh23*^753*A*^) that predispose to early onset, hair cell loss and progressive deafness in mice, thus restricting the progeny that can be used in auditory aging studies and decreasing the utility of this four way cross for auditory aging studies (Noben-Trauth et al., 2003; Mianné et al., 2016). For this reason, earlier studies from our group (Schacht et al., 2012; Altschuler et al., 2015) developed a different four-way cross, UMHET4, in which the grandparent strains lack the *ahl* predisposing Cdh23^753A^ alleles. We returned to use of UMHET4 mice in the current study so that all of the progeny could be used. Our previous studies using UMHET4 mice (Schacht et al., 2012; Altschuler et al., 2015) and pilot animals in the current study showed that UMHET4 mice generally have a later appearing ARHL than UMHET3 mice and that ABR TS do not commonly initiate until around 18 months of age. The current study therefore tested the influence of adding rapamycin to mouse diet at 14 months of age.

## Study Design

UMHET4 mice were entered into the study sequentially as they reached appropriate age points and tested for ABR as they reached 5 months of age. Mice with ABR thresholds more than three standard deviations from the mean at any of the four frequencies (4, 12, 24, and 48 kHz) assessed were excluded from the study. The ABR thresholds at 5 months of age served as individual and group mean baselines and the basis for determining TS at later ages. Mice received a second ABR at 12 months of age and any mice with greater than 10 dB SPL TS at any frequency tested were excluded from the study. At 14 months of age, eligible male and female mice were randomly placed into one of two groups, either a rapamycin diet group or a control diet group. Rapamycin was given encapsulated in the food (pelleted chow) to the mice in the rapamycin treatment group at a dose of 42 mg kg^−1^, the dose found most effective in increasing lifespan by Miller et al. (2014). Both groups had diet changed at 14 months of age, from standard chow to either rapamycin diet or control diet without rapamycin (but containing the other additions to the rapamycin chow that allowed the rapamycin to be encapsulated). Longitudinal ABR measures from mice in both groups were continued at 18 and 24 months of age and TS (from 5 months old baseline) determined. The control diet group started with 37 mice with 29 surviving until euthanasia at 24 months of age and the rapamycin diet group started with 29 mice with 24 surviving until the 24 months old end point. Animals were euthanized within eight days after the 24 months old ABR measure. The left cochleae were used for hair cell count evaluations (cytocochleograms) and the right cochleae were processed and saved for future gene expression studies.

### Breeding

The UM-HET4 mice were generated as described previously (Schacht et al., 2012) by a four-way cross between MOLF/EiJ (Jackson Laboratory stock #000550) × 129S1/SvImJ F1 (Jackson Laboratory stock #002448) female mice and C3H/HeJ (Jackson Laboratory stock #000659) × FVB/NJ F1 (Jackson Laboratory stock #001800) male mice. All of the four grandparental strains lack the *ahl* allele that typically leads to hearing loss appearing at 2–4 months of age (Johnson et al., 2006, for review). Each mouse in the tested UM-HET4 population inherits 25% of its genome from each of the four distinct inbred grandparental stocks and is genetically unique, but shares 50% of its alleles with every other mouse in the tested population. The UM-HET4 mice exhibit variability in their ARHL hearing loss that was correlated with polymorphisms in specific genetic loci (Schacht et al., 2012).

### Auditory Brain Stem Response (ABR)

Animals were anesthetized with ketamine (65 mg/kg), xylazine (7 mg/kg), and acepromazine (2 mg/kg). Body temperature was maintained with water circulating heating pads and heat lamps. Additional anesthetic (ketamine and xylazine) was administered when needed to maintain anesthesia depth sufficient to insure immobilization and relaxation. ABRs were then recorded in an electrically and acoustically shielded chamber (Acoustic Systems, Austin, TX, USA). Needle electrodes were placed at vertex (active), the test ear (reference) and the contralateral ear (ground) pinnae. Tucker Davis Technologies (TDT) System III hardware and SigGen/BioSig software (TDT, Alachua, FL, USA) were used to present the stimulus and record responses. Tones were delivered through a EC1 sound driver (TDT) with the speculum placed just inside the tragus. Stimulus presentation used 15 ms tone bursts, with 1 ms rise/fall times, presented 10 per second. Up to 1,024 responses were averaged for each stimulus level. Responses were collected for stimulus levels in 10 dB steps at higher stimulus levels, with additional 5 dB steps near threshold. Thresholds were interpolated between the lowest stimulus level where a response is observed, and 5 dB lower, where no response is observed. The frequencies tested were 4, 12, 24, and 48 kHz.

### Histology

Mice were euthanized by intraperitoneal injection of Sodium Pentobarbital (Fatal Plus) followed by decapitation. Cochleae were rapidly removed and middle ears assessed for signs of middle ear infection under a dissection microscope. An opening made through the otic capsule in the apex of the left cochleae and fixative (4% paraformaldehyde in phosphate buffer) was slowly infused into the cochlear fluids. The cochleae were then immersed in fixative for 2–6 h at room temperature on a rotator and rinsed in phosphate buffered saline (PBS) before a 16–24 h decalcification in 5% EDTA at room temperature. This was followed by removal of the otic capsule and tectorial membrane. Cochleae were then stained with 1% Phalloidin- Alexa Fluor 568 in PBS and then micro dissected into three segments, apex, base, and hook. Each segment was mounted separately as a surface preparation on a glass slide with Prolong Gold as mounting media. Slides were stored at 4°C before examination and viewing.

### Hair Cell Assessment

Phalloidin labeling of hair cells was used to identify presence or absence of hair cells. Hair cells were counted under epifluorescence optics on a Leica fluorescent microscope under double blind conditions. The number of inner hair cells and outer hair cells (OHC) that were present or absent for each 0.19 mm reticule length was entered into a cytocochleogram program starting at the apex and moving basally until the entire length of the cochlear spiral had been assessed. The program compares hair cell numbers to a normal data base. The program can generate a graph of hair cell loss by position along the cochlear spiral for each cochlea (cytocochleogram), can provide the analysis in absolute numbers, as the total percent of hair cells lost in each animal assessed and can be used to generate means for groups.

### Statistics

Significance for ABR threshold changes was tested using an unpaired “t” test with Welch's correction and the non-parametric Mann–Whitney test with Bonferroni adjustment. Significance was considered at both *p* < 0.01 and *p* < 0.05 levels. There was no significant difference in the mean ABR thresholds or TS between male and females within the rapamycin diet or within the diet control group at any age and so males and females were combined when testing for significance. Means are given in text plus/minus the standard error of the mean (SEM).

## Results

### Auditory Brain Stem Response

**Figure 1:**
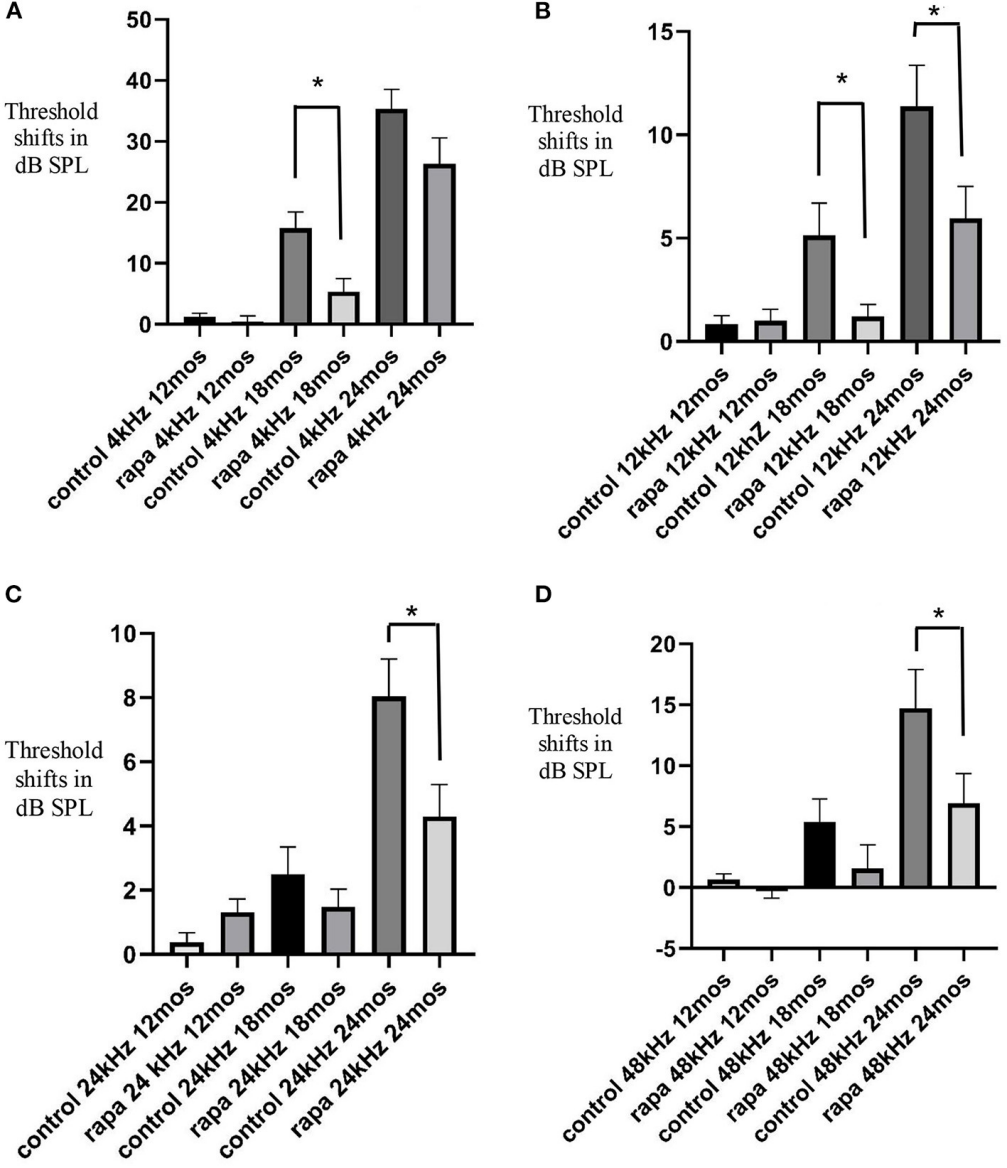
Comparison of mean auditory brain stem response threshold shifts (compared to 5 months of age) in the group with rapamycin added to diet at 14 months of age (rapa) vs. the group on control diet without rapamycin (control) at 12, 18, and 24 months of age at 4 kHz **(A)**, 12 kHz **(B)**, 24 kHz **(C)** and 48 kHz **(D)**. Asterisks indicate significant differences. Please note differences between A, B, C, and D in scale bars for dB SPL on the “y” axis.

**Figure 2:**
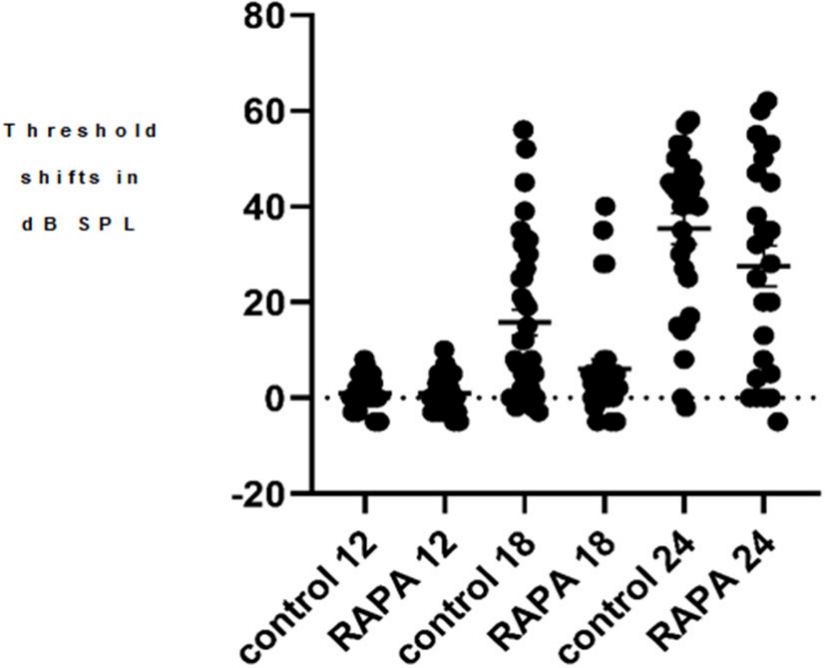
A scatter plot of the 4 kHz auditory brain stem response threshold shifts (compared to 5 months of age) at 12, 18, and 24 months of age in the control diet group (without rapamycin) and in the group with rapamycin added to diet at 14 months of age. There is little variability in either group at 12 months of age. At 18 months of age more variability is seen in the control diet group (control 18) while the rapamycin treatment group (RAPA18) remains more skewed toward little threshold shift. At 24 months of age there is less variability in the control group (control 24) now skewed toward large threshold shifts while the rapamycin treatment group (RAPA24) has more distribution across the range of threshold shifts.

There was no significant difference in the mean ABR thresholds or TS between male and females in either control diet or rapamycin diet groups at any age for any of the frequencies assessed and males and females were therefore combined for comparisons. At 12 months of age there were no animals with TS above 10 dB SPL at any frequency in animals to be assigned to either group. The progression of ABR TS in the control diet group was consistent with the pattern we have previously reported in UMHET4 mice with significant TS first appearing at low frequencies and later at higher frequencies (Schacht et al., 2012; Altschuler et al., 2015) (Figures 1A–D). There was variability across animals in the timing of their progression of hearing loss (e.g., Figure 2 for 4 kHz) with a few mice (3–29) in the control diet group retaining normal hearing thresholds across all frequencies even at the latest 24 months of age ABR assessment time, also consistent with what we have previously observed in UMHET4 mice (Schacht et al., 2012; Altschuler et al., 2015).

### Threshold Shifts at 18 Months of Age

At 18 months of age, the mean ABR TS at 4 kHz in the rapamycin diet group was 5.5 ± 2.2 dB SPL compared to a 15.8 ± 2.7 dB SPL mean in the control diet group, this difference was significant (*p* < 0.01) (Figure 1A). This was a consequence of a larger number of animals (17–37) showing 4 kHz ABR TS over 10 dB in the control diet group compared to 5–29 in the rapamycin diet group. Variability among mice in timing of TS and rapamycin treatment effect is shown in a scatter plot at 4 kHz in Figure 2. At 12 kHz the mean TS in the rapamycin diet group was 1.2 ± 0.6 dB SPL compared to a 5.1 ± 1/6 dB SPL in the control diet group, this difference was also significant (*p* < 0.05). There were no significant differences in 24 or 48 kHz TS means between rapamycin and control diet groups (Figures 1C,D). One mouse in the rapamycin diet group was an outlier with a large (53 dB SPL) TS at 48 kHz without a TS at 4 kHz.

### Threshold Shifts at 24 Months of Age

At 24 months of age there was no longer a significant difference (*p* = 01.4) in the mean 4 kHz TS between the rapamycin diet (27.2 ± 4.5 dB SPL) and the control diet (35.3 ± 3.2 dB SPL) groups (Figure 1A). The difference in mean 12 kHz TS between rapamycin diet (5.9 ± 1.5 dB SPL) and control diet (11.4 ± 2.0 dB SPL) groups remained significant (*p* < 0.05) (Figure 1B). Differences now appeared at higher frequencies, with a significant difference (*p* < 0.05) in mean TS between rapamycin and control diet groups for both 24 kHz (rapamycin = 4.2 ± 1.0 dB SPL; control = 8.0 ± 1.2 dB SPL) and 48 kHz (rapamycin = 6.9 ± 2.5 dB SPL; control = 14.7 ± 3.2 dB SPL) assessments (Figures 1C,D).

### Histology and Hair Cell Assessment

**Figure 3:**
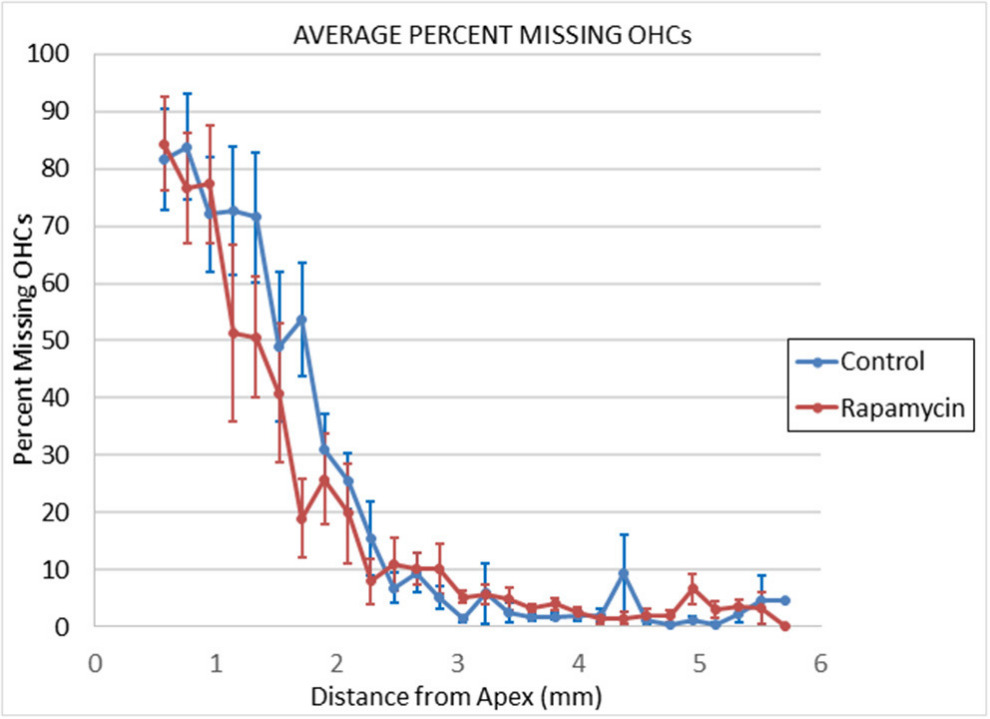
Cytochleograms comparing the mean outer hair cell loss across the cochlea spiral at 24 months of age in mice from the group that had rapamycin added to diet at 14 months of age (red line) vs. the control diet group without rapamycin added to diet (blue line). Apical cochlea is to the left and base to the right, the transition from apical turn to basal turn is ~1.75 mm from the apex and the transition from basal turn to the hook is at ~3.9 mm from apex. There is large loss of outer hair cells in the apical third of the cochlea in both groups and minimal loss in the remaining cochlea (including hook) in both groups.

**Figure 4:**
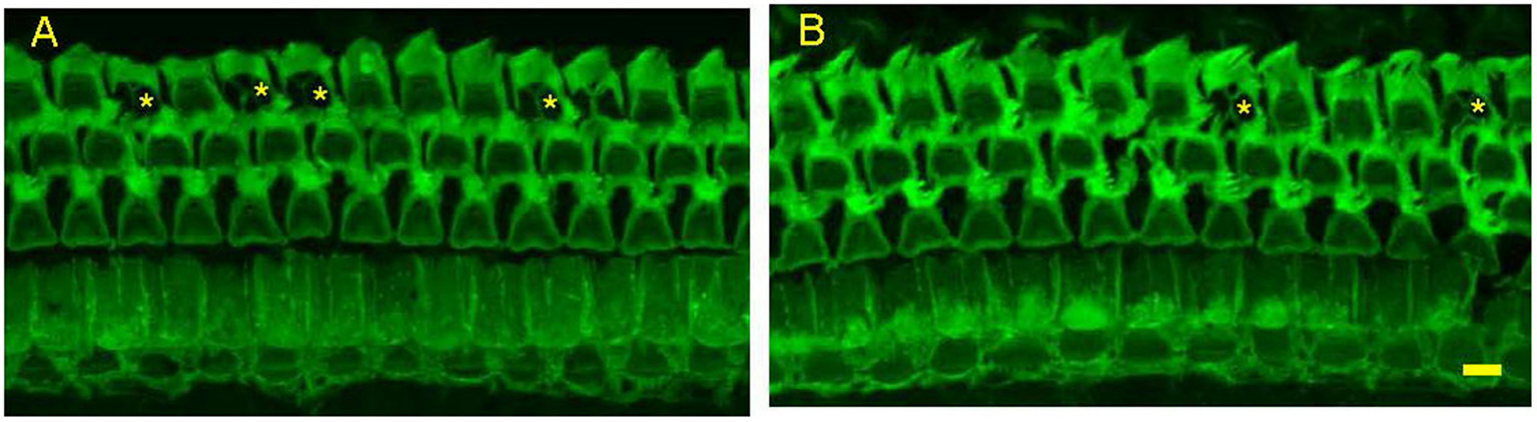
Representative photomicrographs from surface preparations of organ of Corti from a 24 months old Control Diet mouse **(A)** and a 24 months old mouse on the Rapamycin Diet **(B)** Both have outer hair cell loss in the third row, with scars marked by asterisks. Bar = 10 μ.

There were no signs of middle ear infection in the cochleae of any mice in the study. At 24 months of age there was large outer hair cell loss in the apical half of cochlea of most mice in both control diet mice and rapamycin diet groups (Figure 4), with no significant difference in the mean loss between these groups. There was only minimal outer hair cell loss in the basal half of the cochlea of most mice in both groups with mean outer hair cell loss well under 10% in both groups (Figures 3, 4) despite ABR threshold shifts.

## Discussion

The results of the present study extend results of our previous study (Altschuler et al., 2018) that found rapamycin added to diet at 4 months of age reduced outer hair cell loss in the basal half but not apical half of the cochleae of 22 months old UMHET3 mice. The present study used longitudinal measure of ABR to show rapamycin added to diet reduced mean threshold shifts at 4 and 12 kHz in 18 months old UMHET4 mice, largely by reducing the percent of mice showing TS. It is not well understood why hair cell loss occurs first in apical regions, then in basal regions in most mouse models and it has been suggested that different mechanisms could be influencing basal vs. apical age-related hearing loss (e.g., Schulte and Schmiedt, 1992; Dubno et al., 2013; Wu et al., 2020). Since 4 kHz is processed in the apical half of the mouse cochlea, ~1.25 mm from apex (Viberg and Canlon, 2004), the results of the present study show that the influence of rapamycin is not restricted to the basal half of the cochlea. This provides indirect evidence that at least some components underlying age-related hearing loss (those that can be influenced by rapamycin) are present in both basal and apical cochleae. Rapamycin acts on mammalian-target-of-rapamycin (mTOR) pathways (both mTORC1 and mTORC2). These pathways are multifaceted and in turn act on other functional signaling pathways including those associated with metabolism, proliferation, immune response and cell survival (Inoki et al., 2005a,b; Perl, 2015 for reviews; Wataya-Kaneda, 2015). The mTORC1 pathway can influence endoplasmic reticulum (ER) stress and the unfolded protein response (UPR) (e.g., Ye et al., 2015). ER stress-related factors have been shown to increase in the cochleae of aged mice (Wang et al., 2015) and ER stress-mediated apoptosis has been associated with noise-induced, ototoxic drug-induced and age-related hearing losses (Oishi et al., 2015; Wang et al., 2015; Hu et al., 2016; Mahdi et al., 2016). ER stress pathways could therefore be a target of the rapamycin effect on ARHL. Rapamycin could also stimulate the survival pathway of p-Akt (S473) via mTOR2 signaling, including reducing mitochondrial stress. Rapamycin could also act through its influence on the inflammatory response or through inhibition of oxidative stress pathways previously implicated in hair cell pathology (Yamasoba et al., 2013, for review). Future studies will be necessary to identify the specific target or targets and pathways underlying the treatment effect seen in the current study.

Another important result is that the rapamycin treatment-related sparing of 4 kHz hearing loss at 18 months is no longer present at 24 months of age and there is an associated large hair cell loss in the apical cochlea of most rapamycin diet and control diet mice at 24 months of age, with no difference between the groups. This suggests that rapamycin treatment delays but does not prevent hearing loss. It would be valuable to identify the specific mechanisms by which rapamycin delays hair cell loss, not only to increase understanding of general mechanisms underlying ARHL but to determine if the delay could be extended and even turned into prevention. The timing of the last ABR and terminal euthanasia in the present study was before large TS generally occurs at higher frequencies in the UMHET4 mouse model. A greater rapamycin induced sparing of hair cell loss and TS than observed in the current study might therefore be found at a later age when greater hair cell loss is occurring, as observed in the previous study in UMHET3 mice where ARHL occurs more rapidly. The lack of correlation between ABR TS and OHC loss in the more basal cochlea at 24 months of age is consistent with reports of age-related reduction or loss in OHC function with reduced distortion product otoacoustic emissions (DPOAE) appearing before age-related OHC loss (e.g., Syka, 2010, for review). One explanation is an age-related disruption of prestin in morphologically intact OHC (Chen et al., 2009; Syka, 2010). It would be valuable to examine the influence of rapamycin treatment on age-related decrements in DPOAE. The variability in the progression and extent of ARHL seen in the control diet UMHET4 mice may reflect their genetic diversity and we have previously shown this variability can be correlated with polymorphisms in specific genetic loci (Schacht et al., 2012). The variability seen in the treatment effect of rapamycin in the rapamycin diet group might also reflect UMHET4 genetic diversity and it would be interesting to identify such differences in future studies.

The present study also addressed the question of whether beginning rapamycin treatment later in life than the 4 months of age used in Altschuler et al. (2018) would be effective. The results show beginning treatment later in life, at 14 months of age is still effective. This is consistent with studies showing late life rapamycin delivery also enhances life span and delays/reduces age-related declines in cardiac, muscle and cognitive functions (Quarles and Rabinovitch, 2020, for review). The literature also suggests that late life intermittent administration of rapamycin and rapamycin-like compounds (“rapalogs”) with less side-effects in people, can also increase life span and decrease age-related declines (Anisimov et al., 2011; Arriola Apelo et al., 2016; Shavlakadze et al., 2018; Quarles and Rabinovitch, 2020). It would therefore be valuable to test late-life intermittent treatment effects of rapamycin and rapalogs on ARHL.

## Data Availability Statement

The raw data supporting the conclusions of this article will be made available by the authors, without undue reservation.

## Ethics Statement

The animal study was reviewed and approved by VAAAHS Institutional Animal Care and Use Committee.

## Author Contributions

RA, DD, and DK: contributed to the study in design: LK, AK, and CM: carrying out studies and measures: RA, DD, DK, CM, and CS: analysis of results and their impact. All authors contributed to the article and approved the submitted version.

## Conflict of Interest

The authors declare that the research was conducted in the absence of any commercial or financial relationships that could be construed as a potential conflict of interest.
